# Seeking neuroprotection in multiple sclerosis: an ongoing challenge

**DOI:** 10.1172/JCI168595

**Published:** 2023-04-03

**Authors:** Jack P. Antel, Timothy E. Kennedy, Tanja Kuhlmann

**Affiliations:** 1 Montreal Neurological Institute, McGill University, Québec, Canada.; 2 Institute of Neuropathology, University Hospital Münster, Münster, Germany.

## Abstract

Multiple sclerosis (MS) is an autoimmune disease of the CNS, featuring inflammation and demyelination with variable recovery. In this issue of the *JCI*, Kapell, Fazio, and authors address the potential for targeting neuron-oligodendrocyte potassium shuttling at the nodes of Ranvier as a neuroprotective strategy during inflammatory demyelination of the CNS in experimental MS. Their extensive and impressive study could serve as a template for defining the physiologic properties of a putative protective pathway. The authors examined MS features in existent disease models, investigated the impact of pharmacologic intervention, and evaluated its status in tissues from patients with MS. We await future studies that will tackle the challenge of translating these findings into a clinical therapy.

## Early events in multiple sclerosis drive disability

Multiple sclerosis (MS) follows an unpredictable clinical disease course most frequently characterized by initial episodes of neurologic relapses with variable recovery; up to 50%–60% of patients with MS in the pretherapeutic era were subsequently found to develop a progressive increase in neurologic deficits. Genetic factors, including approximately 250 polymorphisms, have been linked to disease susceptibility, yet only now are independent markers linked to progressive disease being identified (1). MS has long been considered an autoimmune disorder, and systemic immune-directed therapies can virtually eliminate clinical relapses, including their MRI counterparts, such as T2 signals or gadolinium-enhancing lesions, collectively referred to as disease activity. Similar therapies when prescribed for the progressive phase have little effect on disease course, independent of the effects on disease activity (2). Based on a review of approximately 200,000 Expanded Disability Status Scale (EDSS) transitions from more than 27,000 patients, Lublin et al. concluded that, although relapses contribute to the accumulation of disability, primarily early on, progression in the absence of disease activity (referred to as PIRA) becomes the principal driver of disability accumulation in the progressive phase of the disease (3).

The dynamic neuropathology of MS further emphasizes the need for early and sustained neuroprotection, as well as repair, and raises concern regarding the timing after which damage becomes irreparable. The acute lesion in MS is characterized by the presence of adaptive immune cells and their products as well as innate constituents. In such lesions, there is evidence of acute axonal transections, but there is still substantial axonal presence (4). The demyelination that defines acute lesions is associated with relative preservation of oligodendrocyte (OL) cell bodies, but OL cell processes die back (5, 6), consistent with such cells being under stress, as confirmed by single-cell molecular analyses of OLs derived from MS lesions (7). Ludwin, as early as 1981, proposed that unraveling of the spiral wrap of myelin was an early event in the development of an MS lesion (8). Spontaneous repair of MS lesions is usually attributed to the recruitment of new OLs that arise from resident OL precursor cells (OPCs) (9). One speculates whether stressed, mature OLs could be rescued and induced to restore axonal wrapping. In more chronic MS lesions, there is progressive loss of OLs and axons; active remyelination is rarely noted (2).

The ongoing challenge in MS is to identify the actual PIRA mechanisms and determine how to target these processes therapeutically. Neurologic disability reflects the loss of nerve impulses reaching their required targets. Meaningful insights require an understanding of the basic physiologic mechanisms that permit and maintain optimal nerve conduction within the CNS and then a determination for how these mechanisms are perturbed in MS. Limitations in animal models that fail to recapitulate the features of progressive MS provide an immediate dilemma in linking physiologic and pathologic states and in translating preclinical findings to the clinic. The most used model of autoimmune-initiated CNS inflammatory and demyelinating disease is experimental autoimmune encephalomyelitis (EAE), which is usually induced in young adult animals by immunization with CNS tissue or myelin peptides over a relatively short period. Toxin models are used to induce acute demyelination, usually with robust spontaneous recovery (9). Targeting specific OL and axon pathways by genetic deletion or pharmacological agents overlooks the complex neuropathology of the CNS in MS, such as the state of glia (including microglia and astrocytes) and extracellular matrices (10, 11).

Advances in imaging and molecular manipulations continue to define the precise physical and molecular properties that underlie OL interactions with axons and allow bidirectional transport of nutrients (reviewed in refs. 12, 13). Myelin critically contributes to organizing the functional domains along axons that are required for saltatory conduction of action potentials. Flanking the node of Ranvier, the layers of compact myelin at the end of a mature internode open to form cytoplasm-filled paranodal loops, establishing specialized septate-like junctions that link the OL to the axon. The paranodal axo-glial junction acts as a molecular fence on either side of the node, which contains densely clustered voltage-gated sodium channels. At the paranode, a cell-cell adhesion complex, composed of the proteins CASPR1 and contactin on the axonal plasma membrane, binds to neurofascin-155 on the OL paranodal loops. The paranode is then flanked by clustered axonal potassium channels in the juxtaparanode.

**Figure 1:**
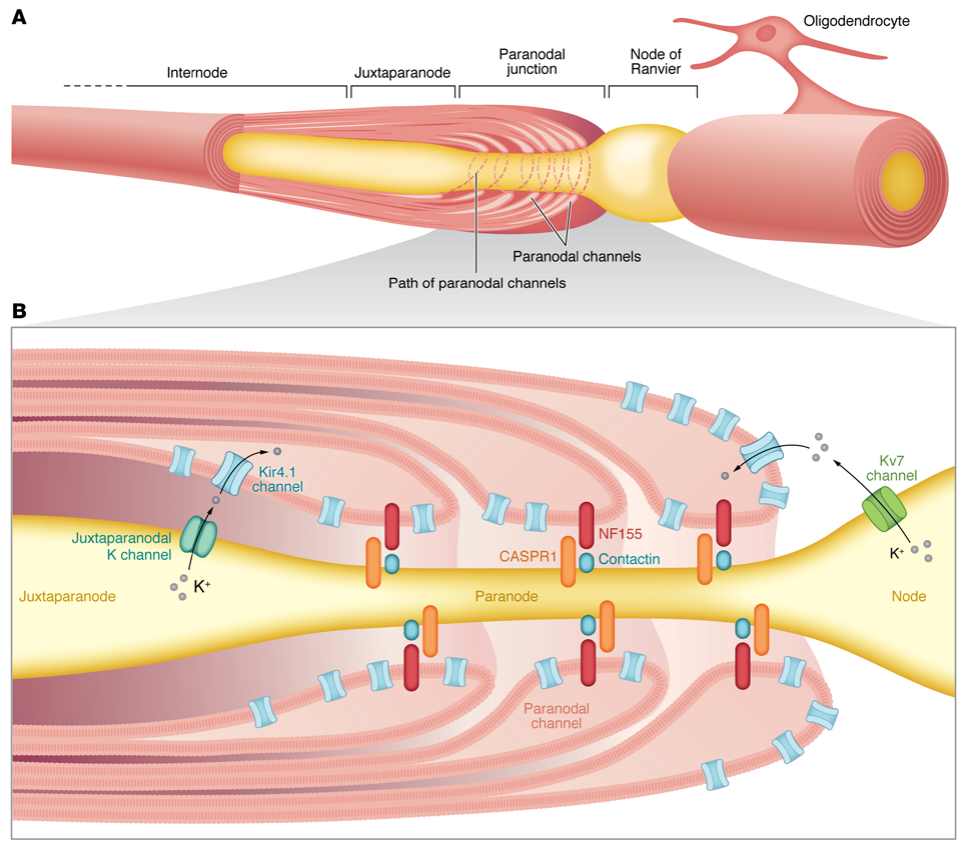
The Kir4.1/Kv7 pathway has a protective role under neuroinflammatory conditions. (**A**) Organization of the specialized axonal domains and oligodendroglial myelin wrapping that flanks the node of Ranvier, including the paranode, juxtaparanode, and internode. (**B**) Neuronal excitability is regulated by outward-rectifying axonal Kv7 potassium channels at the nodes of Ranvier. Inward-rectifying Kir4.1 potassium channels expressed by OLs are localized to the inner tongue, outer tongue, and paranodal loops. Healthy OLs in normal conditions vacuum up the increased extracellular potassium that is released by neuronal activity. Kapell, Fazio, and colleagues report that inflammatory demyelinating conditions result in dysfunction of this neuroglial potassium shuttling mechanism, with downregulation of neuronal Kv7 channels and oligodendroglial Kir4.1 channels. The authors’ findings suggest that targeting potassium channel function to normalize neuronal excitability may prevent neurodegeneration and promote the recovery of function.

In this issue of the *JCI*, Kapell, Fazio, and co-authors show that Kv7.2 potassium channels localize to nodes flanked by CASPR1 in juxtaposition with Kir4.1 on OLs at paranodal loops and inner and outer tongues of noncompacted myelin (Figure 1) (14). Nodal and paranodal regions have been considered particularly vulnerable sites of immune-mediated injury in MS (15). Alterations in these regions can even be observed in normal-appearing white matter (16, 17) and are implicated in contributing to disease progression in MS (17). Ultrastructure changes include uncompacting of myelin, unmasking of potassium channels, and increased axonal energy demand.

## From experimental model to clinical translation

Kapell, Fazio, and co-authors (14) initiated their studies using genetically induced channel-deficient animals in the baseline state and in the EAE model. They used these models to demonstrate the role of Kv7/Kir4.1 neuron-to-OL potassium shuttling in downregulating neuronal hyperactivity and its dysfunction under neuroinflammatory conditions. However, genetic channel deletion would be more akin to human, single-gene inherited disorders. The EAE studies were conducted in animals aged 8–14 weeks, with the longest disease duration, referred to as “chronic EAE,” being 30 days after immunization (14). Still unclear is whether there is an EAE model that can fulfill the criteria of PIRA and in which progression is unresponsive to systemic immune therapy. Also, one should consider the effect of aging, with progressive MS being most recognized in the fifth and sixth decades (18).

Kapell, Fazio, and colleagues (14) applied histologic and molecular analyses to MS tissue samples as a means to bridge the knowledge gap between the preclinical and clinical states. Their immunohistochemical analyses indicated a downregulation of Kir4.1 in acute MS lesions with an upregulation of Kv7.2, suggesting an attempt at compensation. In more chronic MS lesions, such compensation was not maintained. Furthermore, Kir4.1 channel expression by astrocytes contributed to this shuttling, in addition to having effects on the blood-brain barrier. In more chronic MS lesions, the authors noted a loss of OLs, again raising concerns that this protective mechanism may decline as the disease progresses. Notably, OPCs in regions of MS lesions expressed Kir4.1 channels, but there was little evidence that the channels were capable of interacting with axonal targets in the more chronic lesion setting. The rapid progress in spatial transcriptomics should enhance our ability to appreciate changes in cell-cell interactions during an evolving disease state.

The studies that pharmacologically target the neuron-OL potassium shuttling pathway perhaps most pointedly addresses the challenge of clinical translation related to both the efficacy and tolerability of candidate agents. Neuroprotective and regenerative therapies have not yet successfully transitioned from animal models into clinical MS, especially in the context of progressive disease. Examples of such agents include sphingosine-1-phosphate receptor (S1PR) modulators such as fingolimod, which provides protection in toxin models and reduces astrocyte-mediated proinflammatory effects but has no measurable effect in primary progressive MS (reviewed in ref. 19). Other examples of agents that provide protection include siponimod, which is approved for secondary-progressive disease, specifically in patients with MS with active disease, and high-dose biotin (20). The Kv7.2-directed drug retigabine (RTG) (USA name, ezogabine) showed measurable benefits in the EAE model used by Kapell, Fazio, and colleagues (14) that were most apparent when administered prophylactically. RTG was approved for the treatment of partial complex seizures but was later removed from the market by the manufacturer because of limited use (21). RTG use in amyotrophic lateral sclerosis has involved only an 8-week, phase 2 clinical trial, where it reduced motor neuron excitability. Side effects included fatigue and dizziness (22).

The study by Kapell, Fazio, and co-authors (14) also compels us to the search for biomarkers that can serve as an index of disease mechanism and disease progression in MS. Initial studies had shown the presence of antibodies against Kiv4 channels in sera and cerebrospinal fluid (CSF) of patients with MS, but this finding could not be universally confirmed (23). With regard to proteins that contribute to nodal and paranodal regions, proteomics analysis of CSF samples collected from children during their initial presentation of MS identified elevated levels of such proteins, including the paranodal adhesion proteins neurofascin 155 (NF155) and DCC (24). Such findings support the suggestion that noncompacted OL membranes, such as the paranodal loops, may be particularly susceptible to injury and dysfunction.

While preventing, arresting, and reversing the course of progressive MS remains a major unmet challenge, the study by Kapell, Fazio, and colleagues (14) highlights the hopes and concerns that come with translating insightful preclinical observations into effective clinical therapy.

## Version 1 04/03/2023

Electronic publication
